# Robotics in Biomedical and Healthcare Engineering

**DOI:** 10.1155/2017/1610372

**Published:** 2017-08-28

**Authors:** Chengzhi Hu, Qing Shi, Lianqing Liu, Uche Wejinya, Yasuhisa Hasegawa, Yajing Shen

**Affiliations:** ^1^Department of Mechanical and Process Engineering, ETH Zurich, Zurich, Switzerland; ^2^Beijing Institute of Technology, Beijing, China; ^3^Chinese Academy of Sciences, Shenyang, China; ^4^University of Arkansas, Fayetteville, NC, USA; ^5^Nagoya University, Nagoya, Japan; ^6^Mechanical and Biomedical Engineering Department, City University of Hong Kong, Tat Chee Avenue, Kowloon, Hong Kong

The rapid progress of robotic technique provides new opportunities for the biomedical and healthcare engineering. For instance, a micro-nano robot allows us to study the fundamental problems at cellular scale owing to its precise positioning and manipulation ability; the medical robot paves a new way for the low invasive and high efficient clinical operation; and rehabilitation robot is able to improve the rehabilitative efficacy of patients. This special issue aims at exhibiting the latest research achievements, findings, and ideas in the field of robotics in biomedical and healthcare engineering, especially focusing on the upper/lower limb rehabilitation, walking assistive robot, telerobotic surgery, and radiosurgery.

Currently, there is an increasing population of patients suffering from limb motor dysfunction, which can be caused by nerve injuries associated with stroke, traumatic brain injury, or multiple sclerosis. Past studies have demonstrated that highly repetitive movement training can result in improved recovery. The robotic-assisted technique is a novel and rapidly expanding technology in upper/lower limb rehabilitation that can enhance the recovery process and facilitate the restoration of physical function by delivering high-dose and high-intensity training. This special issue covers several interesting papers addressing these challenges. X. Tu and coworkers introduced an upper limb rehabilitation robot powered by pneumatic artificial muscles which cooperates with functional electrical stimulation arrays to realize active reach-to-grasp training for stroke patients. The dynamic models of a pneumatic muscle and functional electrical stimulation-induced muscle are built for reaching training. By using surface electromyography, the subject's active intent can be identified. Finally, grasping and releasing behaviors can be realized by functional electrical stimulation array electrodes. C. Guo and coworkers proposed an impedance-based iterative learning control method to analyze the squatting training of stroke patients in the iterative domain and time domain. Patient's training trajectory can be corrected by integrating the iterative learning control scheme with the value of impedance. In addition, the method can gradually improve the performance of trajectory tracking by learning the past trajectory tracking information and obtain specific training condition of different individuals. The paper demonstrated an effective control methodology in dealing with repeated tracking control problems or periodic disturbance rejection problems. Apart from these works, J. Li and coworkers designed an open-structured treadmill gait trainer for lower limb rehabilitation; T. Sun and coworkers proposed a method for detecting the motion of human lower limbs including all degrees of freedoms via the inertial sensors, which permits analyzing the motion ability according to the rehabilitation needs.

Other biomedical and healthcare robots included in this special issue cover a range of interesting topics, such as walking assistive robot, telerobotic surgery, and radiosurgery. To improve the walking ability of the elderly, the walker-type rehabilitation robot has become a popular research topic over the last decade. C. Tao and coworkers proposed a hierarchical shared control method of the walking-aid robot for both human motion intention recognition and the obstacle emergency-avoidance method based on the artificial potential field. In the implementation, the human motion intention is obtained from the interaction force measurements of the sensory system composed of force sensing registers and a torque sensor. Meanwhile, a laser-range finder forward is applied to detect the obstacles and try to guide the operator based on the repulsion force calculated by artificial potential field. The robot realizes obstacle avoidance while keeping partially the operators' original walking intention. X. Li and coworkers demonstrated a general framework for robot-assisted surgical simulators for a more robust and resilient robotic surgery. They created a hardware-in-the-loop simulator platform and integrated the simulator with a physics engine and a state-of-the-art path planning algorithm to help surgeons acquire an optimal sense of manipulating the robot instrumental arm. Eventually, they achieved autonomous motion of the surgical robot. For coping with the workspace issue during the application of Linac system during radiosurgery, a specialized robotic system was presented by Y. Noh et al. The design and implementation of the robotic system were elaborated. All of these works showed comparative advantages versus classical approaches and will hold great potential for providing insights on the practical and systematic design of robots that serve for broad applications in biomedical and healthcare engineering.

The objectives of the special issue were reached in terms of advancing the state of the art of robotic techniques and addresing the challenging problems in biomedical and healthcare engineering. Several critical problems in these areas were addressed, and most of the proposed contributions showed very promising results that outperform existing studies. Some of the proposed approaches were also validated from patients' perspectives, which show the applicability of these techniques in realistic environments.

